# Smoke-Free Workplaces Are Associated with Protection from Second-Hand Smoke at Homes in Nigeria: Evidence for Population-Level Decisions

**DOI:** 10.1155/2015/618640

**Published:** 2015-10-04

**Authors:** Dorota Kaleta, Kinga Polanska, Bukola Usidame

**Affiliations:** ^1^Department of Tobacco Control, Preventive Medicine, Medical University of Łódź, 90-752 Łódź, Poland; ^2^Department of Public Policy, University of Massachusetts, Boston, MA, USA

## Abstract

The evidence suggests that smoke-free workplace policies may change social norms towards exposing others to second-hand smoke at home. The aim of the study was to assess whether being employed in a smoke-free workplace (SFWP) is associated with living in a smoke-free home (SFH). We used the data from the Global Adult Tobacco Survey conducted in Nigeria in 2012, in which 9,765 individuals were interviewed including 1,856 persons who worked indoors. The percentage of Nigerians employed in SFWP that reported living in a SFH was higher compared to those employed in a workplace where smoking occurred (95% versus 73%). Working in a SFWP was associated with a significantly higher likelihood of living in a SFH (OR = 5.3; *p* < 0.001). Urban inhabitants indicated more frequently that they lived in SFH compared to rural residents (OR = 2.0; *p* = 0.006). The odds of living in a SFH were significantly higher among nonsmokers and nonsmokeless tobacco users compared to smokers and smokeless tobacco users (OR = 28.8; *p* < 0.001; OR = 7.0; *p* < 0.001). These findings support the need for implementation of comprehensive smoke-free policies in Nigeria that result in substantial health benefits.

## 1. Introduction

Taking into account the level of exposure and its health consequences, tobacco smoking and second-hand smoke (SHS) constitute one of the biggest public health threats [1–4]. According to the most recent data, about 40% of children and a third of nonsmoking adults are exposed to SHS [1, 5]. The estimates of worldwide burden of a disease indicated that approximately 600,000 deaths were attributed to SHS with 47% of deaths occurring in women, 28% in children, and 26% in men [5].

Over the years many policies have been implemented in order to improve the health of particular populations [6–9]. In 2008, the World Health Organization (WHO) identified six evidence-based tobacco control measures that are the most effective in reducing exposure to cigarette smoking and environmental tobacco smoke [6]. Among them creation of smoke-free public places and workplaces continues to be the most commonly established measure with the highest level of achievement [10].

Scientific evidence, unequivocally, indicates that there is no safe level of exposure to SHS and that an environment which is completely smoke-free and does not allow any exceptions is the only proven way to fully protect people from the harm of that exposure [2–4, 10]. The increase in the number of countries with comprehensive smoke-free legislation shows that effective laws are relatively easy to pass and enforce and involve little or no cost [10]. This policy measure has high levels of public support, causes no financial harm to businesses, and improves the health of both smokers and nonsmokers. Implementation of smoke-free environments including smoke-free workplaces has been associated with a reduction in tobacco consumption, an increase in smoking cessation, and consequently a reduction in hospital admissions due to cardiovascular and respiratory diseases [11–18]. Moreover, public places smoking bans and home smoking bans are not isolated from each other and do not only protect people from the health risk of SHS but also reduce the likelihood that children will start smoking [11–20].

Despite the existing scientific evidence that creation of 100% smoke-free environments is an effective and inexpensive way of protecting residents from health and economic consequences of tobacco smoke exposure, Nigeria has still not introduced comprehensive smoke-free measures [21–26]. Under provisions of the smoking decree number 20 of 1990, reviewed in 2001, smoking in specific public places such as health-care, educational (except for universities), and governmental facilities is banned in Nigeria [21, 22]. On the other hand, smoke-free environments are still not created and reinforced in the indoor offices, restaurants, cafes, pubs, and bars.

The Global Adult Tobacco Survey (GATS) in Nigeria, as in the first country in Sub-Saharan Africa, provides a special insight into the country's tobacco use and control measures context. Based on the survey estimates in 2012, close to 5 million (5.6%) of Nigerian adults aged 15 years or older used tobacco products and 3 million of them (3.9% of the population) smoked tobacco [22]. Proportion of smokers is higher for men (7.3%) than it is for women (0.4%). In addition, estimated 17.3% of adults (2.7 million people) had been exposed to SHS in their workplaces and 6.6% (5.2 million people) at their homes.

The prevalence of active and passive smoking in Nigeria is not as high as in other low- and middle-income countries but given the high number of smokers and passive smokers together with an increasing trend in tobacco consumption exposure to tobacco smoke is becoming a growing public health threat [22]. The studies performed in Nigeria indicate that despite a high level of awareness of the dangers of SHS and positive attitudes to smoke-free laws, a high proportion of the population is still exposed to SHS in public places, which calls for policy level interventions to improve the implementation of the smoke-free law [23–26]. In addition, the analysis performed in Osun State in Nigeria indicated poor awareness of the existing law of prohibition of smoking in public places which generate the necessity to increase sensitization of the general public [26]. Evaluation of the association between smoke-free public places and SHS exposure at home might be crucial for strengthening implementation and enforcement of the comprehensive legislation prohibiting smoking in public and in workplaces in the country.

The aim of this study was to assess whether being employed in a smoke-free workplace is associated with living in a smoke-free home in Nigeria.

## 2. Materials and Methods

### 2.1. Study Design and Population

Data used for the current analysis is available from the Global Adult Tobacco Survey (GATS), which was conducted in Nigeria in 2012. The complete description of the methodological assumptions has been published elsewhere [22]. Briefly, the survey was designed to generate precise cross-sectional estimates at the national level. The final probability selection of the sample units was equivalent to that of being selected under the three-stage stratified-cluster sampling in order to produce key indicators by gender, for the country as a whole as well as classified by residence (urban or rural), and to allow for comparison of the estimates among the 6 geopolitical zones of Nigeria. Following the GATS sampling protocol, a sample of at least 8,000 respondents was required with 4,000 adults each from urban and rural areas. The household sample size was then adjusted to account for the potential sample size loss due to ineligibility and nonresponse. A total of 11,107 households were sampled, of which 5,776 were from urban areas and 5,331 were from rural areas. One eligible household member was randomly selected from each participating household, which resulted in 9,765 individuals completing the survey. GATS Nigeria included a household questionnaire and an individual questionnaire. The questionnaires were applied during face to face interviews with the persons who were 15 years of age or older and they were recorded on an electronic data collection device.

The overall response rate for GATS Nigeria was 89.1%. The household response rate was 90.3% (86.8% urban, 94.1% rural), while the individual response rate was 98.6% (98.0% urban, 99.2% rural) [22].

Current analysis is restricted to the GATS respondents working indoors or both indoors and outdoors but outside their home (2277 participants). After removing people with missing variables, the final analysis is focused on 1856 people (82% of the population that reported indoor work).

### 2.2. Variables

For the purpose of the current analysis the dependent variable was “living in a smoke-free home.” The participant was classified as living in a smoke-free home if she/he answered “smoking is never allowed inside my home” to the question “Which of the following best describes the rules about smoking inside your home?” If the answer was “smoking is allowed inside my home,” “smoking is generally not allowed inside my home but there are exceptions,” or “there are no rules about smoking at home” he/she was considered as not living in a smoke-free environment. The independent variable was “working in a smoke-free environment” based on answer “no” to the question “During the past 30 days, did anyone smoke in the indoor areas where you work?”

Additional factors included in the analysis were as follows: age (15–29, 30–44, 45–59, 60 years, and above), gender (male, female), number of people in the household, residence (urban, rural), region (North East, North Central, North West, South East, South West, South-South), and education (no formal education, primary school completed, secondary school completed, higher secondary school completed, college/university, and above). Based on the question “Which activity best describes your main work status over the past 12 months?” the participants were divided into the employees (including government and nongovernment employees) and self-employed. If the study subjects indicated current smoking on a daily or less than a daily basis they were considered smokers. Similar classification was considered for smokeless tobacco use (yes, if the participant indicated usage of these products daily or occasionally).

### 2.3. Statistical Analysis

The STATISTICA Windows XP version 10.0 program was used to carry out the statistical analysis. Initially, a descriptive analysis for all the variables involved in the analysis was completed. Univariate and multivariate logistic regression analyses were run to estimate the odds ratio (OR) and 95% confidence intervals (95% CI) of living in a smoke-free home if employed in a smoke-free workplace compared with being employed in a workplace where smoking occurred. The logistic regression model was adjusted for all the covariates (significant at a 0.1 level) to reduce the risk of confounding. Age and number of household members were treated as continuous variables. Analysis is performed for total study population as well as for smokers and nonsmokers separately (see S1–S3 in Supplementary Material available online at http://dx.doi.org/10.1155/2015/618640). We tested for multicollinearity for covariates that were controlled for in the analysis. The multicollinearity diagnostics variance inflation factors (VIF) were all less than five, which indicates that the assumption of reasonable independence among predictor variables was met.

## 3. Results

### 3.1. Characteristics of the Study Participants

Most of the study subjects who worked indoors but out of their homes were younger than 45 (Table 1). Males constituted 60.6% of the population included in analysis. About 66% of the respondents indicated that they completed a higher secondary school or have a college/university degree. Similar percentages of the study participants lived in urban areas. 5.8% of the respondents were current smokers, whereas of the people working indoors, 14.6% indicated SHS exposure in the workplace and 7.9% at home. Among the self-employed nonsmokers, 15.8% indicated exposure to SHS in the workplace, whereas among the nonsmoking employees, 8.2% reported the same (Table 2). For the smokers those percentages were 63.6% among the self-employed and 26.4% among the employees.

### 3.2. Predictors of Smoke-Free Home

Among the Nigerians who reported smoke-free workplaces 95.4% lived in smoke-free homes, whereas among those who indicated SHS exposure in their workplaces smoke-free home was declared by only 73.1% (Table 1). Additional analysis, which was performed for the smokers, indicated that among those who had a smoke-free work environment about half also lived in smoke-free homes (54.2%). The percentage of smoke-free homes was indicated much less frequently by smokers who declared SHS exposure in their workplace (22.4%) (Supplementary Table S1). Among the nonsmokers, smoke-free home was declared by 84.2% of those exposed to SHS in the workplace and 97.0% of those who declared a smoke-free workplace (Supplementary Table S2). The highest proportion of the people who lived in smoke-free homes was observed in the South West region (95.4%) and among the participants with college/university degrees (94.9%). The people who lived in urban areas were more likely to indicate smoke-free homes compared to those from rural areas (94.7% versus 87.1%). The current smokers and smokeless tobacco users were less likely to live in smoke-free homes compared to those who did not indicate any of these habits (39.8% versus 95.4% and 63.6% versus 92.5%, resp.).

Table 3 presents the results of the unadjusted and adjusted logistic regression analyses of the predictors of a smoke-free home. In the univariate model, working in a smoke-free environment was associated with a significantly higher likelihood of living in a smoke-free home (OR = 7.6; *p* < 0.001). This association persisted after including a variety of covariates in the model (OR = 5.3; *p* < 0.001). The analysis performed separately for the smokers and nonsmokers indicated a similar, more than 4 times higher chance of living in a smoke-free home in the case of those working in a smoke-free workplace comparing to the people who declared SHS exposure in the environment of work (among the smokers adjusted OR = 4.4; *p* = 0.005 among the nonsmokers adjusted OR = 4.9; *p* < 0.001) (Supplementary Table S3). Women were significantly more likely to live in a smoke-free home than men (OR = 2.2; *p* < 0.001) in the univariate analysis but not in the multivariate assessment (*p* = 0.8). The people living in urban areas indicated significantly more frequently that they lived in smoke-free homes than those from rural areas in the unadjusted (OR = 2.6; *p* < 0.001) and similarly in the adjusted analyses (OR = 2.0; *p* = 0.006). In the univariate analysis the chance of having smoke-free homes was higher in the South West region (OR = 2.5; *p* = 0.002), but higher in the North West Nigeria in the case of the multivariate logistic regression (OR = 2.3; *p* = 0.05). In the univariate analysis, more respondents with a higher level of education indicated that smoking was never allowed inside their homes compared to those that have completed secondary school education (higher secondary school OR = 2.4; *p* = 0.007; college/university OR = 3.0; *p* = 0.003). However, in the multivariate analysis, those that have completed a primary school (OR = 3.9; *p* = 0.003) and a higher secondary school (OR = 2.9; *p* = 0.005) had a higher chance of living in smoke-free homes. The odds of living in a smoke-free home were significantly higher for the nonsmokers and the nonsmokeless tobacco users relative to those who indicated they were current smokers and smokeless tobacco users (OR = 31.1; *p* < 0.001; OR = 7.0; *p* < 0.001 resp.). The multivariate results confirmed the figures observed in the univariate analysis (OR = 28.8; *p* < 0.001; OR = 7.0; *p* < 0.001, resp.).

## 4. Discussion

The study indicated that working in a smoke-free workplace was associated with a significantly higher likelihood of living in a smoke-free home after adjusting for a variety of confounders. In addition, the people living in urban areas as well as the nonsmokers and nonsmokeless tobacco users indicated significantly more frequently that they lived in smoke-free homes than those from rural areas, current smokers, and smokeless tobacco users.

The analysis, which utilized data from the GATS conducted in 15 low- and middle-income countries between 2008 and 2011, indicated, similarly as our assessment, positive associations between being employed in a smoke-free workplace with living in a smoke-free home (for 13 countries such associations were statistically significant) [11]. However, substantial differences in the percentage of people indicating both smoke-free homes and smoke-free workplaces have been observed between the countries (varied from 21% in China to 75% in Mexico). In Nigeria, such percentages were even higher than those observed in Mexico, Thailand (73%), and Ukraine (71%). This can result from the low prevalence of smoking observed in Nigeria compared to the other countries. In addition, some cultural and religious norms or high level of awareness about the dangers of exposure to SHS, as well as evidence of support for tobacco control laws, could be responsible for the observed results [11, 12, 23–26]. Similar results (as observed in low- or middle-income countries) are indicated in longitudinal studies performed in the US, where living in a country fully covered by a 100% clean indoor air law in workplaces or restaurants/bars was associated with an increased likelihood of having a voluntary 100% smoke-free home rule both for people living with smokers (OR = 7.8, 95% CI = 5.3–11.4) and not living with smokers (OR = 4.1, 95% CI = 3.3–5.2) [27, 28]. Comparably, significant reduction in smoking at home after implementation of comprehensive smoke-free policies has been observed in Ireland and in the UK [29]. Another evaluation by Edwards et al. (2008) has indicated that, in New Zealand between 2003 and 2006, SHS exposure in workplaces decreased from 20% to 8% and proportion of smoke-free homes increased from 64% to 70% [30].

These results provide evidence against arguments that smoke-free legislation may displace smoking from public to private places and can be used as the tool for implementing 100% smoke-free public places in Nigeria. It needs to be stressed that in 2004 Nigeria signed and in 2005 ratified the WHO FCTC, which highlighted the importance of accelerating the implementation of comprehensive tobacco control legislation in this country [21]. The other aspect, which needs to be considered, is that although the percentages of active and passive smokers in this country are not as high as in other low/middle-income countries, taking into account the extent of the population this constitutes a significant public health problem. This means that implementing comprehensive policy measures might result in significant benefits.

A smoke-free workplace is a cost-effective, public health approach that encourages the important long-term goal of eliminating tobacco use and SHS exposure [7, 9]. The existing evidence indicates that creation of public and private policies to restrict smoking has been found to be an effective approach to promoting cessation including reduction of the average daily consumption of cigarettes, increasing the percentage of smokers contemplating quitting, and increasing the percentage of successful quitting [13–18]. This public health approach can affect large numbers of individuals at minimal cost and thus is an essential component of any successful strategy to promote smoking cessation.

Our results, similar to most low- or middle-income countries, indicate that the people in urban settings were more likely to live in a smoke-free home environment than those from rural areas. This can be explained by different types of dwellings observed in these two areas (of enclosed structure in urban and open space in rural settings) [11]. In addition, the rural areas have the highest level of illiteracy in the country, which might also explain the higher prevalence of tobacco use and SHS exposure [31].

Our results show that there is a higher chance of having smoke-free homes in the North West compared to the North Central region of Nigeria. Data from the GATS Nigeria (for the whole population included in the survey) indicated that the residents of the North Central region (12.6%; 1.3 million) had the highest and those from the North West (3.6%; 0.6 million) the lowest SHS exposure at home among all regions [11]. The differences between the regions can result from sociodemographic, cultural/religious, and economic determinants as well as from the implementation of policy measures and public awareness about the active and passive smoking and their consequences [11].

In the current analysis the self-employed individuals constitute about 60% of the population and in this group, among smokers as well as nonsmokers, the exposure to SHS in the workplace was indicated more frequently than among the employees. The case of a self-employed person still follows the logic that even if a person works away from home, as a one-man business, he or she has no motivation to set a smoke-free policy only for him/herself. It can be assumed that if the study had included the average number of employees in the workplace in the data collection, it would have provided a clearer understanding of the issue of limited smoke-free workplace among the self-employed. This requires further attention and indicates that such a group of workers constitutes the target group for antismoking and policy interventions.

Not surprisingly, the nonsmokers and nonsmokeless tobacco users indicated significantly more frequently that they lived in smoke-free homes than those who declared current smoking status and smokeless tobacco use.

The study has several strengths. The Global Adult Tobacco Survey (GATS) is a cross-sectional, nationally representative survey and covers a large number of respondents obtained from a general population framework, and so it ensures the reliability and validity of the results. In addition, the data obtained for the current analysis are based on similar questions as those used for the assessment performed in other low- and middle-income countries, which guarantees direct comparability of the results [11, 12].

The limitations of the study also need to be considered. Firstly, the lack of verification of self-reported smoking status and SHS exposure at home/workplace can create misclassification. The verification of active or passive smoking by biomarkers or environmental measurements is generally not feasible for the large cross-sectional studies. However, studies indicate that validated self-reported smoking status and SHS exposure are accurate in most studies and correlate well with the biomarker measurements [32]. Secondly, the cross-sectional study in which both variables (smoke-free workplace and smoke-free home) are measured simultaneously limits causal interpretation of our findings. However, the longitudinal studies conducted in other countries have demonstrated that people who worked in smoke-free workplaces are more likely to live in smoke-free homes [27–30]. Poor surveillance of tobacco use in Nigeria means that more robust prepost or longitudinal study designs applied to explain the association cannot yet be employed.

In Nigeria, in the future studies, questions on the most effective and appropriate interventions for different sectors of the workforce (such as men and women, younger and older workers, temporary or casual workers) need to be addressed. Moreover, there is a need to identify the most effective ways of encouraging employee compliance with a smoke-free policy and resource needs of the large, medium, and small enterprises in implementing smoke-free legislation. Based on the policy recommendations for Nigeria the adaptation and implementation of the law must be a collaborative effort between federal, state, and local governments [33].

## 5. Conclusions

Our results support the evidence that smoke-free workplaces have the important additional effect of stimulating smoke-free homes in Nigeria. Since home remains a major source of SHS exposure for children, this work clearly indicates additional justification for enacting smoke-free workplaces as the motivation for voluntary smoke-free home rules. The results from the GATS can also be used against arguments that a smoke-free legislation may displace smoking from public to private places and strengthen implementation of the 100% smoke-free legislation in Nigeria.

## Supplementary Material

The tables S1-S3 presents a descriptive statistics for respondent characteristics as well as predictors of smoke-free home performed for smokers and nonsmokers separately. The percentage of smoke-free homes was indicated much less frequently by smokers who declared SHS exposure in their workplace (22.4%) (Table S1). Among the nonsmokers, smoke-free home was declared by 84.2% of those exposed to SHS in the workplace and 97.0% of those who declared a smoke-free workplace (Table S2). More than 4 times higher chance of living in a smoke-free home in the case of those working in a smoke-free workplace comparing to the people who declared SHS exposure in the environment of work was observed (among the smokers adjusted OR = 4.4; *p* = 0.005 among the nonsmokers adjusted OR = 4.9; *p* < 0.001) (Table S3).

## Figures and Tables

**Table 1:** Descriptive statistics for the respondent characteristics.

	Respondents (employee or self-employed) who worked indoors (out of home) *N* = 1856			
	Total respondents who worked indoors		Smoke-free at home	
	*N*	%	*N*	%
Smoke-free in the workplace	1585	85.4	1512	95.4
SHS in the workplace	271	14.6	198	73.1
Age				
15–29	478	25.7	439	91.8
30–44	895	48.2	820	91.6
45–59	365	19.7	342	93.7
60 and above	118	6.4	109	92.4
Gender				
Male	1125	60.6	1014	90.1
Female	731	39.4	696	95.2
Residence				
Rural	622	33.5	542	87.1
Urban	1234	66.5	1168	94.7
Geographical regions				
North East	141	7.6	122	86.5
North Central	206	11.1	184	89.3
North West	253	13.6	225	88.9
South East	266	14.3	239	89.9
South West	718	38.7	685	95.4
South-South	272	14.7	255	93.8
Education				
No formal education	326	17.6	284	87.1
Primary completed	209	11.3	193	92.3
Secondary school completed	100	5.4	86	86.0
Higher secondary school completed	852	45.9	797	93.5
College/university and above	369	19.9	350	94.9
Occupation				
Employee	770	41.5	719	93.4
Self-employed	1086	58.5	991	91.3
Current smoking				
Yes	108	5.8	43	39.8
No	1748	94.2	1667	95.4
Smokeless tobacco use				
Yes	22	1.2	14	63.6
No	1834	98.8	1696	92.5

SHS: second-hand smoke.

**Table 2:** Second-hand smoke (SHS) exposure among the self-employed and employees depending on their smoking status.

SHS exposure in the workplace	Self-employed *N* = 1086		Employee *N* = 770	
	Smokers *n* = 55	Nonsmokers *n* = 1031	Smokers *n* = 53	Nonsmokers *n* = 717
No	20 (36.4%)	868 (84.2%)	39 (73.6%)	658 (91.8%)
Yes	35 (63.6)	163 (15.8%)	14 (26.4%)	59 (8.2%)

**Table 3:** Predictors of a smoke-free home. Crude and adjusted odds ratio (95% CI).

	Crude	*p*	Adjusted	*p*
	OR (95% CI)		OR (95% CI)	
Smoke-free in the workplace				
Yes	7.6 (5.3–10.9)	*p* < 0.001	5.3 (3.4–8.5)	*p* < 0.001
No	1 (ref.)		1 (ref.)	
Age (years)	1.0 (0.99–1.0)	*p* = 0.4		
Gender				
Male	1 (ref.)		1 (ref.)	
Female	2.2 (1.5–3.2)	*p* < 0.001	0.9 (0.6–1.5)	*p* = 0.8
Residence				
Rural	1 (ref.)		1 (ref.)	
Urban	2.6 (1.9–3.7)	*p* < 0.001	2.0 (1.2–3.2)	*p* = 0.006
Regions				
North East	0.8 (0.4–1.5)	*p* = 0.4	0.7 (0.3–1.5)	*p* = 0.3
North Central	1 (ref.)		1 (ref.)	
North West	1.0 (0.5–1.7)	*p* = 0.9	2.3 (1.0–5.2)	*p* = 0.05
South East	1.1 (0.6–1.9)	*p* = 0.9	0.9 (0.4–1.9)	*p* = 0.8
South West	2.5 (1.4–4.4)	*p* = 0.002	1.2 (0.6–2.5)	*p* = 0.7
South-South	1.8 (0.9–3.5)	*p* = 0.08	1.1 (0.5–2.6)	*p* = 0.8
Education				
No formal education	1.1 (0.6–2.1)	*p* = 0.8	2.2 (1.0–4.9)	*p* = 0.05
Primary completed	2.0 (0.9–4.2)	*p* = 0.08	3.9 (1.6–9.7)	*p* = 0.003
Secondary school completed	1 (ref.)		1 (ref.)	
Higher secondary school completed	2.4 (1.3–4.4)	*p* = 0.007	2.9 (1.4–6.1)	*p* = 0.005
College/university and above	3.0 (1.5–6.2)	*p* = 0.003	2.3 (0.9–5.6)	*p* = 0.07
Occupation				
Employee	1.4 (1.0–1.9)	*p* = 0.1	1.3 (0.8–2.2)	*p* = 0.9
Self-employed	1 (ref.)		1 (ref.)	
Current smoking				
Yes	1 (ref.)		1 (ref.)	
No	31.1 (19.9–48.6)	*p* < 0.001	28.8 (16.8–49.5)	*p* < 0.001
Smokeless tobacco use				
Yes	1 (ref.)		1 (ref.)	
No	7.0 (2.9–17.0)	*p* < 0.001	7.0 (2.5–19.3)	*p* < 0.001
Number of people in household	0.99 (0.9–1.0)	*p* = 0.7		
