# Supplementary material for: Smoke-Free Workplaces Are Associated with Protection from Second-Hand Smoke at Homes in Nigeria: Evidence for Population-Level Decisions
Source: Biomed Res Int. 2015 Oct 4;2015:618640. doi: 10.1155/2015/618640 (PMC4609351; doi:10.1155/2015/618640)
Supplement: Supplementary file 1 — The tables S1-S3 presents a descriptive statistics for respondent characteristics as well as predictors of smoke-free home performed for smokers and nonsmokers separately. The percentage of smoke-free homes was indicated much less frequently by smokers who declared SHS exposure in their workplace (22.4%) (Table S1). Among the nonsmokers, smoke-free home was declared by 84.2% of those exposed to SHS in the workplace and 97.0% of those who declared a smoke-free workplace (Table S2). More than 4 times higher chance of living in a smoke-free home in the case of those working in a smoke-free workplace comparing to the people who declared SHS exposure in the environment of work was observed (among the smokers adjusted OR = 4.4; p = 0.005 among the nonsmokers adjusted OR = 4.9; p < 0.001) (Table S3). [file 618640.f1.pdf]

Smoke-free workplaces are associated with protection from second hand smoke at homes in Nigeria - evidence for population-level decisions

Dorota Kaleta[1*], Kinga Polanska[1], Bukola Usiadame[2]

[1] Department of Tobacco Control, Preventive Medicine, Medical University of Łódź, Łódź, Poland

[2] Department of Public Policy, University of Massachusetts, Boston, US

Supplementary materials

The tables S1-S3 presents a descriptive statistics for respondent characteristics as well as predictors of smoke-free home performed for smokers and nonsmokers separately. The percentage of smoke-free homes was indicated much less frequently by smokers who declared SHS exposure in their workplace (22.4%) (Table S1). Among the nonsmokers, smoke-free home was declared by 84.2% of those exposed to SHS in the workplace and 97.0% of those who declared a smoke-free workplace (Table S2). More than 4 times higher chance of living in a smoke-free home in the case of those working in a smoke-free workplace comparing to the people who declared SHS exposure in the environment of work was observed (among the smokers adjusted OR = 4.4; p = 0.005among the nonsmokers adjusted OR = 4.9; p < 0.001) (Table S3).

Table S1. Descriptive statistics for respondent characteristics - smokers

	Respondents (employee or self-employed) who worked indoors (out of home) N=108			
	Total respondents who worked indoors		Smoke-free at home	
	N	%	N	%
Smoke-free in workplace	59	54.6	32	54.2
SHS in workplace	49	45.4	11	22.4
Age				
15-29	20	18.5	10	50.0
30-44	59	54.6	21	35.6
45-59	21	19.4	9	42.9
60 and above	8	7.4	3	37.5
Gender				
Male	107	99.1	42	39.3
Female	1	0.9	1	100.0
Residence				
Rural	55	50.9	15	27.3
Urban	53	49.1	28	52.8
Geographical regions				
North East	12	11.1	3	25.0
North Central	15	13.9	2	13.3
North West	26	24.1	13	50.0
South East	16	14.8	5	31.3
South West	25	23.1	15	60.0
South South	14	13.0	5	35.7
Education				
No formal education	28	25.9	12	42.9
Primary completed	15	13.9	6	40.0
Secondary school completed	3	2.8	0	0.0
Higher secondary school completed	46	42.6	18	39.1
College/university and above	16	14.8	7	43.8
Occupation				
Employee	53	49.1	21	39.6
Self-employed	55	50.9	22	40.0
Smokeless tobacco use				
Yes	1	0.9	0	0.0
No	107	99.1	43	40.2

SHS- Second hand smoke

Table S2. Descriptive statistics for respondent characteristics – non--smokers

	Respondents (employee or self-employed) who worked indoors (out of home) N=1748			
	Total respondents who worked indoors		Smoke-free at home	
	N	%	N	%
Smoke-free in workplace	1526	87.3	1480	97.0
SHS in workplace	222	12.7	187	84.2
Age				
15-29	458	26.2	429	93.7
30-44	836	47.8	799	95.6
45-59	344	19.7	333	96.8
60 and above	110	6.3	106	96.4
Gender				
Male	1018	58.2	972	95.5
Female	730	41.8	695	95.2
Residence				
Rural	567	32.4	527	92.9
Urban	1181	67.6	1140	96.5
Geographical regions				
North East	129	7.4	119	92.2
North Central	191	10.9	182	95.3
North West	227	13.0	212	93.4
South East	250	14.3	234	93.6
South West	693	39.6	670	96.7
South-South	258	14.8	250	96.9
Education				
No formal education	298	17.0	272	91.3
Primary completed	194	11.1	187	96.4
Secondary school completed	97	5.6	86	88.7
Higher secondary school completed	806	46.1	779	96.7
College/university and above	353	20.2	343	97.2
Occupation				
Employee	717	41.0	698	97.4
Self-employed	1031	59.0	969	94.0
Smokeless tobacco use				
Yes	21	1.2	14	67.7
No	1727	98.8	1653	95.7

SHS- Second hand smoke

Table S3. Predictors of smoke-free home. Crude and adjusted odds ratio (95% CI) - analysis for smokers and non-smokers

	Smokers				Non-smokers			
	Crude OR (95% CI)	p	Adjusted OR (95% CI)	p	Crude OR (95% CI)	P	Adjusted OR (95% CI)	p
Smoke-free at workplace								
Yes	4.1 (1.7-9.6)	p=0.001	4.4 (1.6-12.2)	p=0.005	6.0 (3.8-9.6)	p<0.001	4.9 (3.0-8.0)	p<0.001
No	1 (ref.)		1 (ref.)		1 (ref.)		1 (ref.)	
Age (years)	1.0 (1.0-1.03)	p=0.9			1.02 (1.00-1.04)	p=0.04	1.0 (1.0-1.1)	p=0.01
Gender*								
Male					1 (ref.)			
Female					0.9 (0.6-1.5)	p=0.8		
Residence								
Rural	1 (ref.)		1 (ref.)		1 (ref.)		1 (ref.)	
Urban	3.0 (1.3-6.7)	p=0.009	3.8 (1.2-12.1)	p=0.02	2.1 (1.3-3.3)	p=0.001	1.4 (0.3-2.4)	p=0.2
Regions								
North East	2.2 (0.3-16.1)	p=0.4	1.6(0.2-13.9)	p=0.7	0.6 (0.2-1.5)	p=0.3		
North Central	1 (ref.)		1 (ref)		1 (ref.)			
North West	6.5 (1.2-35.5)	p=0.03	13.3 (1.9-93.6)	p=0.01	0.7 (0.3-1.6)	p=0.4		
South East	3.0 (0.5-18.8)	p=0.2	1.7 (0.2-12.3)	p=0.6	0.7 (0.3-1.7)	p=0.4		
South West	9.8 (1.8-54.1)	p=0.01	3.9 (0.6-24.9)	p=0.2	1.4 (0.7-3.2)	p=0.4		
South-South	3.6 (0.6-23.4)	p=0.2	2.7 (0.4-20.0)	p=0.3	1.5 (0.6-4.1)	p=0.4		
Education								
No formal education	1.3 (0.5-3.4)	p=0.6	1.5 (0.7-3.2)		1.3 (0.3-2.8)	p=0.4	1.4 (0.6-3.4)	p=0.4
Primary completed	1.1 (0.3-3.8)	p=0.8	2.2 (0.96-5.2)		3.4 (1.3-9.1)	p=0.01	3.1 (1.1-8.9)	p=0.03
Secondary school completed **	1 (ref.)				1 (ref.)		1 (ref.)	
Higher secondary school completed					3.7 (1.8-7.7)	p<0.001	2.4 (1.1-5.4)	p=0.03
College/university and above	1.3 (0.4-4.3)	p=0.6			4.4 (1.8-10.7)	p=0.001	1.7 (0.6-4.9)	p=0.3
Occupation								
Employee	0.98 (0.5-2.1)	p=0.9			2.4 (1.4-4.0)	p=0.001	1.6 (0.8-3.0)	p=0.2
Self employed	1 (ref.)				1 (ref.)		1 (ref.)	
Smokeless tobacco use***								
Yes					1 (ref.)		1 (ref.)	
No					11.2 (4.4-28.5)	p<0.001	9.4 (2.1-28.7)	p=0.001
Number of people in household	1.1 (0.9-1.2)	p=0.2			0.95 (0.9-1.01)	p=0.1	1.1 (0.9-1.1)	p=0.3

*gender was not included in analysis among smokers (as only 1 women smoked cigarettes)

**among smokers the reference variable was created as combined variables: „secondary school completed" and „higher secondary school completed' (as only 3 smokers indicated "secondary school completed").

*** variable "smokeless tobacco use" was not included in analysis among smokers (as only 1 person indicated the usage of smokeless tobacco)
